# Impacts of Social Capital Factors on Blood Glucose Control and Depressive Symptoms

**DOI:** 10.1093/geroni/igab046.2387

**Published:** 2021-12-17

**Authors:** Tingzhong (Michelle) Xue, Zahra Rahmaty, Eleanor McConnell, Yingzhi (Lindsay) Xu, Kirsten Corazzini

**Affiliations:** 1 Duke University School of Nursing, Durham, North Carolina, United States; 2 University of Maryland School of Nursing, Baltimore, Maryland, United States; 3 Duke University, Durham, North Carolina, United States

## Abstract

Social capital, conceptualized as resources arising from social networks, is receiving increased attention for its role in prevention and management of chronic conditions such as diabetes and depression that commonly co-occur. Although social capital has been linked to control of blood glucose and depression, previous research has not considered these two outcomes simultaneously while distinguishing between cognitive (i.e., perceived social support, shared values and trust in community) and structural (i.e., social connectedness and participation) domains. This study examined how these two domains of social capital relate to glucose control and depressive symptoms, and whether physical exercise and care access mediate those relationships, using structural equation modeling. The sample included 3,043 older adults aged 57 and above from wave 2 of the National Social Life, Health and Aging Project. Although a higher level of cognitive social capital was associated with higher levels of physical exercise (b=.38, p<.001), access to care (b=.40, p=.007), lower levels of blood glucose (b=-.43, p<.001) and depressive symptoms (b=-.84, p<.001), a higher level of structural social capital was associated only with a higher level of physical exercise (b=.16, p=.002). The mediating effects of physical exercise and access to care were not significant. Findings suggest that cognitive social capital may have greater influence on blood glucose and depressive symptoms than structural social capital, and therefore have different implications for practice, especially in the context of pandemic-related disruptions to social capital. Future research should examine other mediators and investigate how promotion of cognitive social capital might improve health outcomes.

